# Development and validation of a new predictive model for in-hospital postoperative major adverse cardiovascular and cerebrovascular events after general anesthesia in nonagenarians undergoing non-cardiac surgery

**DOI:** 10.3389/fcvm.2025.1590496

**Published:** 2025-06-10

**Authors:** Lan Feng, Xuemei Tan, Xiaoxia Duan, Jiang Zheng, Xiaohui Du, Hong Fu, Yu Ma

**Affiliations:** ^1^ Department of Anesthesiology, Chongqing Emergency Medical Center, Chongqing University Central Hospital, School of Medicine, Chongqing University, Chongqing, China; ^2^ Department of Anesthesiology, Chongqing General Hospital, Chongqing University, Chongqing, China; ^3^ Department of Anesthesiology, The Affiliated Hospital of Southwest Medical University, Luzhou, Sichuan, China; ^4^ Department of Intensive Care Unit, Chongqing Emergency Medical Center, Chongqing University Central Hospital, School of Medicine, Chongqing University, Chongqing, China

**Keywords:** major adverse cardiovascular events, cerebrovascular events, nomogram, prediction model, aged

## Abstract

**Background:**

Major adverse cardiac and cerebrovascular events (MACCE) following noncardiac surgery are the main cause of perioperative mortality. However, there are few evidence-based prediction models available for predicting the risk of MACCE. We aimed to analyze the risk factors of MACCE in patients aged 90 and older and to construct a prediction model, ultimately leading to the development of a nomogram.

**Methods:**

This review study included clinical data from 872 patients aged 90 and older who underwent non-cardiac surgery under general anesthesia between 2015 and 2024. The outcome of interest was in-hospital postoperative MACCE. Logistic regression was employed to identify risk factors and to establish a nomogram for predicting the risk of MACCE. Calibration curves, C-index, and decision curves were used to evaluate the predictive model. An external cohort was used to compare the performance between our model and the widely used revised cardiac risk index (RCRI) score.

**Results:**

112 patients (12.84%) experienced in-hospital MACCE. The final model identified four predictors, including emergency surgery, neutrophil/lymphocyte ratio (NLR) ≥ 11.2, D-dimer ≥ 3.6 mg/L, and postoperative admission to the ICU. The nomogram demonstrated strong discriminative ability with a C statistic of 0.853 and maintained its performance during 10-fold cross-validation with a C statistic of 0.784. Compared to the RCRI score, our predictive model performed better in the validation test (C statistic = 0.853 vs. 0.693).

**Conclusions:**

The predictors including NLR, D-dimer, emergency surgery, postoperative 24-hour ICU admission could better predict MACCE than RCRI score in patients greater than 90 years old undergoing non-cardiac surgery undergoing general anesthesia.

## 1 Introduction

An increasing number of elderly patients are undergoing surgery and anesthesia (1). Many elderly patients present with preoperative frailty or cardiovascular/cerebrovascular comorbidities, increasing their risk of postoperative adverse events following noncardiac surgery (2). And these adverse events are leading cause of morbidity and mortality after noncardiac surgery (3).

Major adverse cardiovascular and cerebrovascular events (MACCE) are defined as in-hospital mortality, acute myocardial infarction (AMI), or ischemic stroke (2). It has been reported that approximately one-third of elderly patients experienced MACCE after noncardiac surgery (4). Advanced age is often deemed as a risk factor to surgery as advancing age often correlates with increased co-morbidities and frailty (5). However, it is important to realize that the elderly, as a cohort, are not a homogenous group of patients (6). Compared to younger patients, older patients have a higher mortality and incidence of complications after noncardiac surgery (7). Compared to patients aged 60–89, patients aged 90 and older had a significantly increased risk of cardiovascular and cerebrovascular complications and mortality after hip replacement surgery (8). Nonagenarians represent a special group of people because of their advanced age and significantly increased risk of perioperative adverse events. Moreover, a cohort study found that undergoing general anesthesia was an independent risk factor for postoperative MACCE in noncardiac surgery patients with age over 18 years (4). Our previous study also found that undergoing general anesthesia (OR, 3.31, 95%CI: 1.91–5.76, *P* < 0.001) was an independent risk factors for 30-day hospital mortality and serious postoperative complications in patients aged 90 years and older (9). Nonagenarians were considered at extremely high risk of adverse events after general anesthesia. It is of great necessity to predict the risk of in-hospital postoperative MACCE in this population, and therefore provide a basis for clinical decision-making.

Accurate preoperative identification of patients at risk for postoperative MACCE is essential in clinical practice. However, reliable tools for determining which patients require intensified observation after surgery are limited, and there is uncertainty surrounding the most effective risk stratification model. Currently, among the various indexes available to estimate risk for MACCE after noncardiac surgery, the Revised Cardiac Risk Index (RCRI) (10) and the National Surgical Quality Improvement Program (NSQIP) risk model (11) developed by the American College of Surgeons are usually recommended by guidelines (12, 13). Among them, RCRI is the most widely validated and used model (14). However, the construction of the RCRI model is based on patients aged 50 and older with a wide age span, and mainly concentrated on cardiovascular adverse events, which has certain limitations in clinical application (10). With respect to the NSQIP model, the real risk of perioperative myocardial infarction is underestimated (15), and none of the NSQIP-derived calculators have been robustly externally validated (16, 17). Moreover, it is too complicated to use at the bedside (18). To date, no research team has focused on individuals aged 90 and older to establish a prediction model for in-hospital postoperative MACCE following noncardiac surgery.

Our team leveraged the hospital's resources to establish a new clinical prediction model for in-hospital postoperative MACCE specifically for this unique population. We recruited patients aged 90 years and older who are undergoing non-cardiac surgery under general anesthesia and explored risk factors for MACCE. We aimed to identify patients who are at higher risk of experiencing in-hospital postoperative MACCE across all surgical procedures. This would allow us to take timely actions to reduce perioperative risks for those classified as high-risk for MACCE. Risk stratification enables us to be better prepared to respond to emergencies and complications that may arise during the perioperative period. On the other hand, timely communication with patients and their families about the expected risks before surgery can effectively reduce conflicts between healthcare providers and patients, thereby improving the doctor-patient relationship. This is the primary significance of this study.

## 2 Methods

### 2.1 Design and ethical approval

It was a multicenter, restrospective observational study conducted in accordance with the Transparent Reporting of a Multivariable Prediction Model for Individual Prognosis or Diagnosis guidelines (TRIPOD) (19, 20). It was registered in the Chinese Clinical Trial Center (registration number ChiCTR2400081240, available online at https://www.chictr.org.cn/) and approved by the Ethics Review Committee of three large-scale comprehensive hospitals.

### 2.2 Study setting and population

We conducted retrospective review of all patients who underwent in-hospital noncardiac surgeries with age over 90 years by electronic health records system in the three large-scale comprehensive hospitals (including Chongqing University Central Hospital, Chongqing General Hospital and The Affiliated Hospital of Southwest Medical University) between January 2015 to January 2024.

### 2.3 Eligibility criteria

The inclusion criteria were as follows: age ≥90 years; American Society of Anesthesiologists (ASA) grade I–III; New York Heart Association (NYHA) class I–II; noncardiac surgery; endotracheal intubation general anesthesia. The exclusion criteria were as follows: preoperatively recently occurred adverse cardiovascular events, including sudden cardiac arrest, non-fatal heart failure, arrhythmias with hemodynamic abnormalities, cardiogenic shock, myocardial injury, or within 4–6 weeks after acute myocardial infarction, and within 3 months after a stroke; incomplete data.

### 2.4 Variables and data collection

Trained clinical reviewers collected clinical data from the medical chart, operative archives, anesthesia records, and progress notes. We collected demographic data, preoperative co-morbidities, laboratory results, and perioperative data, including age, gender, Barthel Index (BI) score (especially evaluating for frailty), ASA classification, emergency surgery, medical history (including hypertension, diabetes, coronary heart disease, arrhythmia, heart failure; dementia, epilepsy, cerebral infarction, cerebral hemorrhage; Chronic Obstructive Pulmonary Disease (COPD), asthma, pneumonia, bronchiectasis, pulmonary embolism; renal insufficiency), preoperative RCRI score, patient's last preoperative laboratory results before surgery, including C-reactive protein (CRP), interleukin-6 (IL-6), neutrophil/lymphocyte ratio (NLR), platelet (PLT), hemoglobin (Hb), albumin (Alb), D-dimer, operation time, intraoperative blood loss, blood transfusion, use of vasoactive drugs, total length of hospital stays, postoperative length of hospital stays, postoperative new-onset MACCE, postoperative admission to intensive care unit (ICU) within 24 h, in-hospital mortality, hospitalization expenses.

### 2.5 Outcomes

The primary outcome was the incidence of in inpatient ward MACCE after noncardiac surgery. The MACCE were diagnosed by cardiologist, including the composite outcomes of in-hospital all-cause mortality, AMI, or stroke, also including cardiogenic shock, and cardiac arrest (2). The secondary outcome were total length of hospital stay and total inpatient costs.

### 2.6 Statistical analyses

Statistical analysis was performed using R4.3.1 (Packages including foreign, rms, rmda, readxl) and SPSS 26.0. Continuous variables conformed to the normal distribution were presented as the mean ± standard deviation, and t-test was used for comparison between two groups. While medians (Interquartile range) and Wilcoxon tests were used for continuous variables not conformed to normal distribution. Categorical variables were presented as percentages with Chi-squared test or Fisher's exact test for comparison between two groups. The cutoff values for continuous data were calculated using receiver operating characteristic curves (ROC) and transformed into binary variables. Missing data for indicators within 10% were supplemented using multiple imputation method. *P* value <0.05 was considered statistically significant for differences.

We combined data from three medical centers into one complete dataset and then randomly divided the entire data set into two parts, including the training set and the validation set. We developed the prediction model using the training set and then tested it with the validation set. Variables with *p* < 0.1 in univariate analysis, as well as the recognized risk factors for MACCE (age ≥ 75, preoperative comorbidities, including hypertension, diabetes, coronary heart disease (21) and previous stroke (22) were further incorporated into multivariate logistic regression analysis. Backward stepwise method was used to choose variables ultimately be included in the model. The variables included in the model were used to construct the nomogram using the package “rms”. We used odds ratio (OR) as the weight to assign a value to each variable. The area under the curve (AUC) for the receiver operating characteristic (ROC) curve was used to validate the discrimination efficiency of the model and the calibration curve was used to assess the model's calibration. In addition, we further use 10-fold cross-validation method to divide all dataset into 10 equally sized sample sets, with a ratio of 1:9. We then refitted the model to each of the 10 datasets, which contained 90% of all data, and calculated the AUC for the unused 10% in each time. Finally, we took the average of all the AUCs to comprehensively evaluate the performance of this model.

## 3 Results

**Figure 1:**
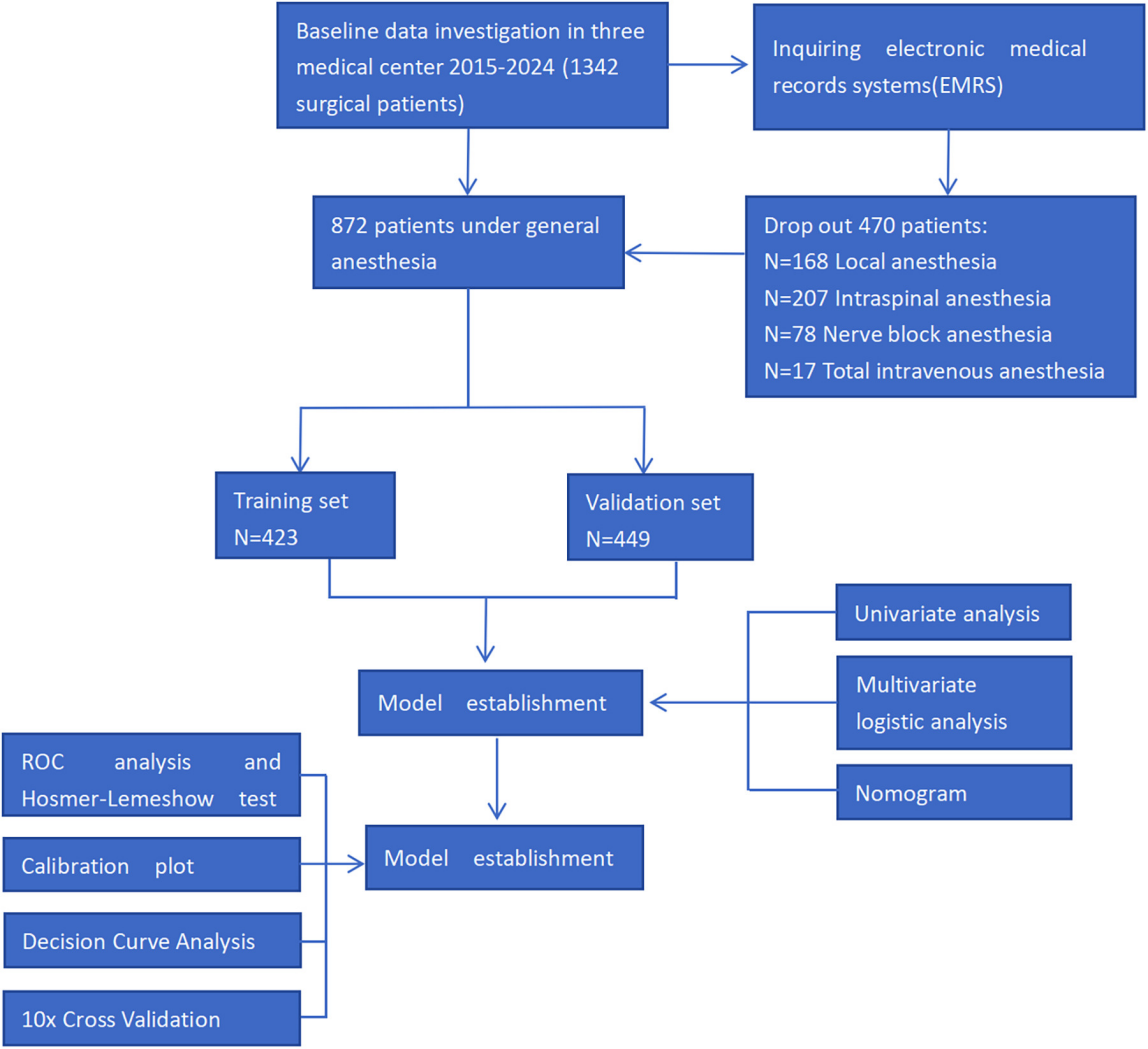
Study flow diagram.

This study initially recruited a total of 1,342 noncardiac surgical patients aged 90 years and older from three medical centers. We used patients' data from either two of three hospitals as training set (*N* = 423), and patients' data from the other hospital as validation test (*N* = 449) to establish a clinical prediction model for MACCE in super-elderly. Figure 1 displayed the flow diagram. A total of 872 patients aged 90 and older (with an average age of 92.61, and 45.61% male) were ultimately included in this study. 112 patients (12.84%) experienced MACCE. Among these cases, 34 patients experienced AMI, six patients experienced cardiac arrest, 26 patients experienced cardiogenic shock or heart failure, 26 patients experienced stroke, and 46 patients died (see Supplementary Table S1). The average total length of hospital stay for patients aged 90 and older was 18 days, with an average post noncardiac surgery hospital stay of 13 days. The average total hospitalization costs for patients aged 90 and older undergoing noncardiac surgery was 6372 ± 156 USD. By drawing ROC curves to transform all continuous variables into binary, the results of ROC curves showed that variables including age, BI score etc, had no predictive power, as shown in Supplementary Table S2.

In the training group, there were a total of 423 patients, of whom 75 (17.7%) experienced MACCE. In the validation group, 37 out of 449 patients experienced MACCE, accounting for 8.2%. The training group exhibited significantly higher rates of MACCE, ICU admission. The average total length of hospital stay was longer and hospitalization costs was much higher when compared to the validation group, as shown in Table 1.

**Table 1:** Comparison of clinical data between training group and validation group.

Indictors	Training set (*n* = 423)	Validation set (*n* = 449)	*P*
Age (years, M ± SD)	92.3 ± 2.4	92.9 ± 2.9	0.002
Male (*n*, %)	186 (44.0%)	213 (47.4%)	0.304
ASA grade			<0.001
II	22 (5.2%)	80 (17.8%)	
III	283 (66.9%)	273 (60.8%)	
	112 (26.5%)	94 (20.9%)	
V	6 (1.4%)	2 (0.4%)	
Emergency (*n*, %)	103 (24.3%)	190 (42.3%)	<0.001
Underlying disease (*n*, %)			
Hypertension	215 (50.8%)	198 (44.1%)	0.047
Coronary heart disease	187 (44.2%)	160 (35.6%)	0.010
Heart failure	54 (12.8%)	64 (14.3%)	0.521
Cardiac arrhythmia	187 (44.2%)	63 (14.0%)	<0.001
Diabetes	61 (14.4%)	58 (12.9%)	0.518
COPD	67 (15.8%)	177 (39.4%)	<0.001
Asthma	5 (1.2%)	8 (1.8%)	0.465
Pneumonia	109 (25.8%)	107 (23.8%)	0.508
PE	0 (0.0%)	5 (1.1%)	0.062
Bronchiectasis	81 (19.1%)	66 (14.7%)	0.079
Dementia	38 (9.0%)	15 (3.3%)	<0.001
Seizure	5 (1.2%)	5 (1.1%)	0.924
Stroke	132 (31.2%)	62 (13.8%)	<0.001
Kidney disease	61 (14.4%)	61 (13.6%)	0.722
Preoperative lab results			
NLR ≥ 11.2	224 (53.0%)	199 (44.3%)	0.011
Hb ≤ 112 (g/L)	251 (59.3%)	208 (46.3%)	<0.001
Na ≤ 140 (mmol/L)	266 (62.9%)	264 (58.8%)	0.217
D-dimer ≥ 3.6 (mg/L)	132 (31.2%)	39 (8.7%)	<0.001
Alb ≤ 34.7 (g/L)	181 (42.8%)	123 (27.4%)	<0.001
Introperative data			
Blood loss ≥ 110 (ml)	216 (51.1%)	36 (8.0%)	<0.001
Blood transfusion (*n*, %)	169 (40.0%)	24 (5.3%)	<0.001
ICU admission	229 (54.1%)	130 (29.0%)	<0.001
Outcomes (*n*, %)			
MACCE	75 (17.7%)	37 (8.2%)	<0.001
MI	7	4	0.312
Cardiac arrest	6	4	0.465
Cardiac shock	24	12	0.026
Stroke	20	16	0.388
Death	36 (8.5%)	10 (2.2%)	<0.001
Total inpatient costs (RMB) (M ± SD)	57,695.42 ± 1,768.75	35,634.94 ± 1,255.97	<0.001
In hospital (days) (M ± SD)			
Totle	24.89 ± 17.11	12.20 ± 7.49	<0.001
After surgery	18.46 ± 14.27	8.42 ± 6.15	<0.001

COPD, chronic obstructive pulmonary disease; PE, pulmonary embolism; NLR, neutrophil-to-lymphocyte ratio; MACCE, major adverse cardiovascular and cerebrovascular events; MI, myocardial infarction.

Univariate analysis in training group showed significant differences in emergency surgery, heart failure, arrhythmia, NLR ≥ 11.2, Hb ≤ 112 (g/L), D-dimer ≥ 3.6 (mg/L), Alb ≤ 34.7 (g/L), and ICU admission rate between the MACCE and NMACCE patients (*P* < 0.05), as shown in Supplementary Table S3. Variables with *P* < 0.1 in the univariate analysis as well as the recognized risk factors for MACCE were further included in the multivariate logistic regression analysis, and the results showed that emergency surgery (OR = 2.652,95%CI: 1.364–5.156,*P* = 0.004), NLR ≥ 11.2 (OR = 2.566, 95%CI: 1.303–5.051,*P* = 0.006c), D-dimer ≥ 3.6 mg/L (OR = 2.175, 95%CI: 1.237–3.825,*P* = 0.007), and ICU admission (OR = 3.089, 95%CI: 1.557–6.128,*P* = 0.001) were independent risk factors for MACCE in patients aged 90 and older after general anesthesia, as shown in Table 2.

**Table 2:** Multivariate analysis of postoperative MACCE.

Indicators	OR	95%CI	*P*
ASA grade			0.824
I	reference		
II	1.430	0.300–6.822	0.654
III	1.401	0.275–7.141	0.685
IV	3.146	0.277–35.744	0.355
Emergency	2.652	1.364–5.156	0.004
Heart Failure	2.090	0.969–4.508	0.060
Arrhythmia	1.055	0.561–1.985	0.868
Dementia	1.653	0.724–3.776	0.233
NLR ≥ 11.2	2.566	1.303–5.051	0.006
Hb ≤ 112 (g/L)	1.538	0.825–2.869	0.176
Na ≤ 140 (mmol/L)	1.143	0.627–2.085	0.662
D-dimer ≥ 3.6 (mg/L)	2.175	1.237–3.825	0.007
Alb ≤ 34.7 (g/L)	1.563	0.879–2.778	0.128
ICU Admission	3.089	1.557–6.128	0.001

Hb, hemoglobin; ALB, albumin; NLR, neutrophil-to-lymphocyte ratio.

**Figure 2:**
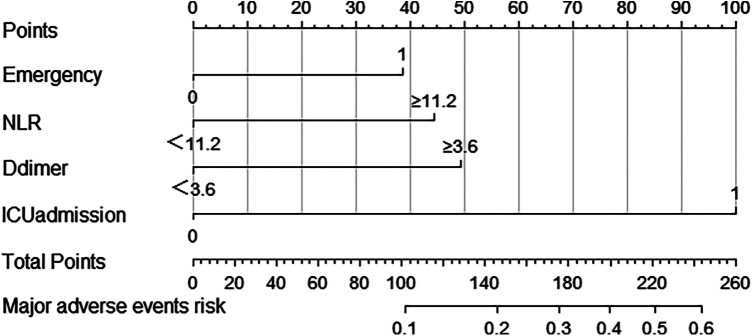
Nomogram for in-hospital postoperative MACCE after noncardiac surgery in patients aged 90 and older under general anesthesia.

After incorporating the aforementioned risk factors into multivariable logistic regression model, the predictive probability PRE_1 of this model was automatically generated. By analyzing the new variable PRE_1, the ROC curve was plotted for this model (see Supplementary Figure S1), with an AUC of 0.752 (95% CI: 0.692–0.812, *P* < 0.001). The Hosmer-Lemeshow test for this model indicated that there was no statistically significant difference between the predicted and the observed values (chi-square = 3.651, *P* = 0.724). We further utilized the model to draw a nomogram (see Figure 2) and a calibration curve (see Supplementary Figure S2), and calculated the total score after scoring each risk factor, in order to evaluate the risk of MACCE more intuitively. The calibration curve was close to the diagonal, indicating a good fitness between the actual risk and the predicted risk. And the C-index was 0.752, suggesting a good predictive performance of the model.

**Figure 3:**
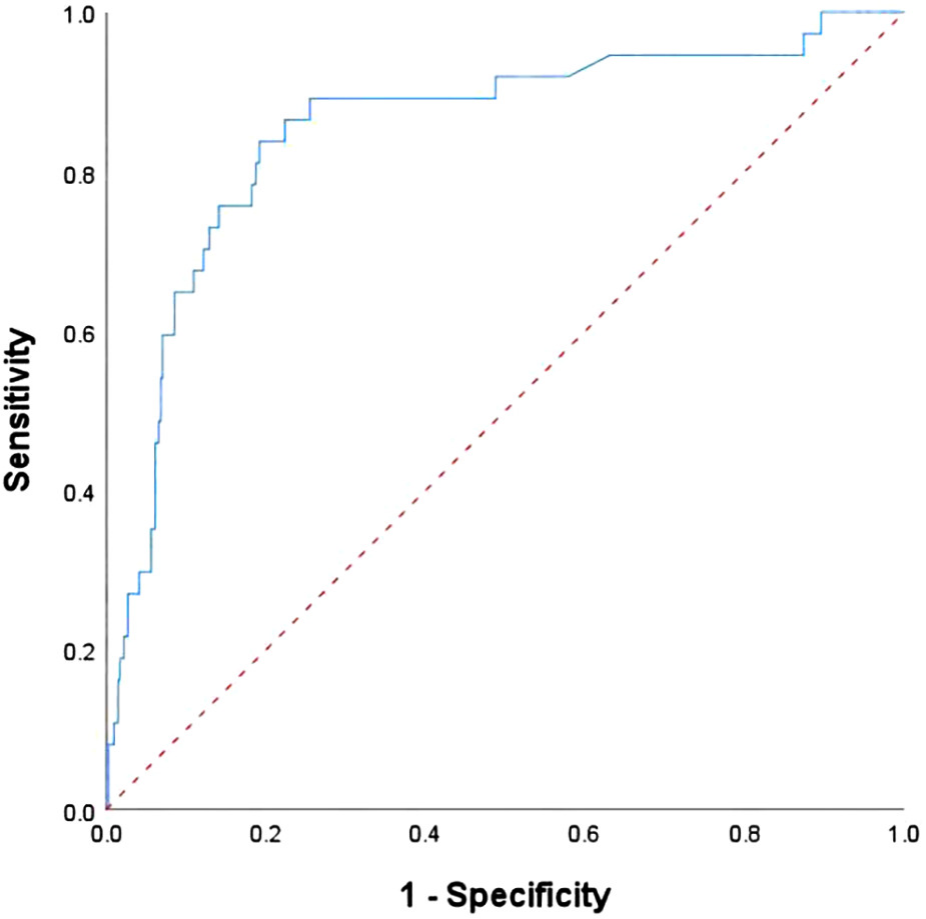
ROC curve for the risk prediction model A of postoperative MACCE in validation group.

With respect to discrimination in the validation test, our prediction model A (C statistic, 0.853; 95%CI: 0.781–0.925, *P* < 0.001) performed well in the validation test, as shown in Figure 3. The Hosmer-Lemeshow test showed that there was no statistically significant difference between the predicted and the observed values (*χ*^2^ = 6.437, *P* = 0.598). Based on external validation, this study also performed internal 10-fold cross-validation. The results showed that the average AUC was 0.784 (0.687–0.951), which was similar to the results of the training set, as shown in Supplementary Table S4. To further validate the predictive power of our nomogram, we assessed the RCRI scoring model using the data from the validation test, and we found that the performance of RCRI (C statistic, 0.693; 95%CI: 0.591–0.795) was relatively poor, as shown in Supplementary Figure S3. In addition, decision curve analysis was widely used to evaluate the clinical value of nomogram. As shown in Supplementary Figure S4, the nomogram demonstrated a significant positive net benefit from the risk of MACCE.

Since the clinical prediction model A we constructed included the indicator of postoperative 24 h ICU admission, which might limit its clinical application by being unsuitable for guiding preoperative decision-making, we reconstructed prediction model B after removing the postoperative 24 h ICU admission indicator. The results showed an area under the ROC curve of 0.781 (95%CI: 0.705, 0.857, *P* < 0.001), as shown in Supplementary Figure S5. The Hosmer-Lemeshow test showed that there was no statistically significant difference between the predicted and the observed values (*χ*^2^ = 12.376, *P* = 0.135).

## 4 Discussion

With the rapid advancement of medical technology and reforms, the aging population has become increasingly prominent, leading to a significant rise in the proportion of elderly patients undergoing surgery, especially those with cardiovascular diseases (23). We performed this retrospective analysis on 872 patients aged 90 and older, and found that the incidence of postoperative in-hospital MACCE was 12.84%, slightly lower than the incidence of MACCE in patients with coronary heart disease (14.3%) (24). This may be related to the relatively low proportion of patients with coronary heart disease in our study cohort. We validated the RCRI score using the validation test, and the results indicated that its discriminatory efficiency was poor (C statistic: 0.693; 95% CI: 0.591–0.795). This suggests that the RCRI score may not effectively differentiate risk in patients aged 90 and older. Therefore, there is an urgent need for a perioperative risk model that can address the increasing number of patients aged 90 and older requiring surgery in China.

We developed and validated a simple risk assessment tool for the individualized perioperative prediction of MACCE in hospitalized patients preparing to undergo noncardiac surgery. Our prediction model incorporated four risk factors, including emergency surgery, NLR (Neutrophil-to-lymphocyte ratio) ≥11.2, D-dimer ≥3.6 (mg/L), and postoperative ICU admission. Current guidelines recommend the use of biomarkers in perioperative evaluations, including N-terminal pro-B-type natriuretic peptide (NT-pro BNP) and high-sensitivity cardiac troponin (25). However, incorporating these biomarkers into routine screening could lead to a significant waste of medical resources. Our model includes leukocyte count, lymphocyte count, and D-dimer levels, all of which can be routinely screened before surgery in China. This approach aims to assess perioperative risk while making efficient use of available medical resources.

It is well known that preoperative assessments and preparations for emergency surgeries are often inadequate, and that preexisting comorbidities may be unstable (26). The risks associated with surgery and anesthesia are extremely high, and the prognosis is often not as favorable as that of elective surgeries (27). Many studies have shown that undergoing emergency surgery significantly increases the risk of MACCE and death during the patient's postoperative hospital stay (28, 29). Consistent with those studies, this also applies to nonagenarians. Our findings indicated that undergoing emergency surgery significantly increased the risk of postoperative MACCE in patients aged 90 and older. Therefore, a thorough evaluation of the timing of surgery, along with proper preoperative preparation, is essential for these patients. The ratio of neutrophils to lymphocytes (NLR) is an emerging inflammatory marker that is closely associated with the relationship between the immune system and diseases (30). It can reflect the severity of diseases and the prognosis of patients (30). Our findings indicated that preoperative NLR exceeding 11.2 were associated with postoperative in-hospital MACCE and enhanced risk prediction in patients aged 90 and older undergoing noncardiac surgery, aligning with previous long-term prospective clinical trials. Elevated NLR is associated with postoperative MACCE during hospitalization (27) and long-term mortality (30) as demonstrated in clinical studies. In addition, NLR could also independently predict the risk of MACCE in diabetic patients (31) and hypertensive inpatients aged 80 and older (32). NLR could be used as inexpensive and broadly available tools for perioperative MACCE risk assessment. Another indictor, D-dimer is the product of hypercoagulable state and secondary fibrinolytic activity in the body (33), which helps identify patients at high risk of thrombosis (34) and cerebrovascular (35) or cardiovascular event (36). It has been reported that elevated plasma D-dimer levels could predict postoperative heart failure in patients undergoing percutaneous coronary intervention (PCI) (37). Consistently, our study found that preoperative elevated D-dimer levels significantly increased the risk of postoperative MACCE in patients aged 90 and older, with a threshold value of 3.6 mg/L. D-dimer levels may serve as a potential biomarker for predicting postoperative in-hospital MACCE in nonagenarians. In clinical practice, patients admitted to the ICU postoperatively, whether following elective or emergency surgery, often experience debility and critical illness (38). Patients admitted to the ICU after surgery have a much higher probability of experiencing MACCE compared to other patients (39). Our results aligned with this finding, suggesting that postoperative ICU admission was an independent risk factor for developing MACCE in patients aged 90 and older. Through re-analysis of the data, we discovered that preoperative cardiovascular and cerebrovascular diseases accounted for a higher proportion of patients admitted to the ICU. Additionally, ICU admission may serve as a mediating factor, significantly increasing the risk of postoperative MACCE. Therefore, the early identification and medical intervention of preoperative cardiovascular and cerebrovascular diseases may significantly reduce the occurrence of MACCE, thereby improving patient prognosis and decreasing perioperative mortality.

Different from other studies (40), our study did not find correlation between ASA classification and the occurrence of MACCE. This may be attributed to the subjectivity and non-uniform nature of ASA classification (41). The same patient evaluated by different anesthesiologists may be categorized into different ASA grades. Relying solely on this simple and rough ASA classification could not accurately predict patients' outcomes (42). Some previous studies found that much older patients had much higher risks of developing MACCE after undergoing noncardiac surgery (43, 44). But in this study, the patients we included were all aged 90 and older, with a narrow age span, and the influence of age on MACCE was excluded in this population. Therefore age was not an independent risk factor for postoperative MACCE in our study.

RCRI is a simple and easy-to-use perioperative MACCE risk scoring tool, primarily designed to assess whether the perioperative MACCE risk is elevated in non-elderly patients with no prior history of cardiovascular and cerebrovascular diseases undergoing non-cardiac surgery (45). According to recent studies on the Chinese population, the predictive value of RCRI for cardiovascular and cerebrovascular events has limitations in high-risk patients, particularly for those with existing cardiovascular and cerebrovascular diseases (4) and older patients (46). In this study, we conducted an external validation of the RCRI score for patients aged 90 and older, yielding a C statistic of 0.693. In contrast, the prediction model we developed showed improved discrimination, achieving a C statistic of 0.752 in the training set and 0.853 in the validation set, both significantly better than the RCRI (*P* < 0.05). We visualize these data using nomogram, which is conducive to clinicians' risk judgement and targeted treatment. To improve our model's applicability, we used multicenter data to validate the nomogram through internal and external validation. Surprisingly, the nomogram showed satisfatory prective value not only in training and internal validation cohort but also in external validation cohort. Importantly, this clinical prediction model was specifically designed for patients aged 90 years and older, taking into account the aging population in China and the current state of medical technology.

Our study also has some limitations. Firstly, our Model A can only be served as a postoperative risk stratification tool incorporating ICU admission status, rather than a preoperative decision-making aid. Its primary clinical utility lies in identifying high-risk surgical patients requiring intensified monitoring or customized interventions during the critical first 24 postoperative hours. Additionally, as a retrospective study, some valuable indicators for predicting postoperative MACCE, such as metabolic equivalents, were not routinely recorded in the medical documentation. We did not follow up on the occurrence of MACCE in patients who left the hospital without seeking further consultation, which may have led to an underestimation of the observed rate of postoperative MACCE. Lastly, our MACCE prediction model was developed using retrospective data, future large-scale prospective studies are necessary to further validate its predictive efficacy and applicability.

## 5 Conclusions

In conclusion, we developed a nomogram that demonstrates superior predictive performance compared to the RCRI in patients aged 90 and older undergoing noncardiac surgery, making it a potentially valuable tool at the bedside. Future large-scale prospective studies are essential to further validate its predictive efficacy and applicability.

## Data Availability

The raw data supporting the conclusions of this article will be made available by the authors, without undue reservation.
