# Supplementary material for: Development and validation of a new predictive model for in-hospital postoperative major adverse cardiovascular and cerebrovascular events after general anesthesia in nonagenarians undergoing non-cardiac surgery
Source: Front Cardiovasc Med. 2025 Jun 10;12:1590496. doi: 10.3389/fcvm.2025.1590496 (PMC12185435; doi:10.3389/fcvm.2025.1590496)
Supplement: Supplementary file 1 [file Supplementaryfile1.docx]

Supplementary Material

**1 SUPPLEMENTARY TABLES**

**Supplementary Table 1.** Characteristics of 872 non-cardiac surgical patients aged 90 and older.

| Characteristic | Values | |
| --- | --- | --- |
| Age(years, M±SD) | 92.61±2.67 | |
| Male(n,%) | 398(45.64%) | |
| BI score(M,IQR) | 45.76(20.83) | |
| RCRI score(M,IQR) | 1.03(1.01) | |
| ASA grade(n,%) |  | |
| II | 102(11.70%) | |
| III | 556(63.76%) | |
| IV | 206(23.62%) | |
| V | 8(0.92%) | |
| Emergency(n,%) | 292(33.49%) | |
| Underlying disease(n,%) | | |
| Hypertension | | 413(47.36%) |
| Coronary heart disease | | 347(39.79%) |
| Heart failure | | 118(13.53%) |
| Cardiac arrhythmia | | 251(28.78%) |
| Diebetes | | 119(13.65%) |
| COPD | | 244(27.98%) |
| Asthma | | 13(1.49%) |
| Pneumonia | | 216(24.77%) |
| PE | | 5(0.57%) |
| Bronchiectasis | | 147(16.86%) |
| Dementia | | 53(6.08%) |
| Seizure | | 10(1.15%) |
| Stroke | | 195(22.36%) |
| Kidney disease | | 122(13.99%) |
| Preoperative lab results | | |
| WBC(10^3^/uL) (M±SD) | | 8.56±4.08 |
| NLR(M,IQR) | | 10.64(6.84) |
| Hb(g/L) (M±SD) | | 110.07±21.77 |
| Plt(10^3^/uL) (M±SD) | | 182.43±77.45 |
| Na(mmol/L) (M±SD) | | 139.33±4.82 |
| K(mmol/L) (M±SD) | | 3.98±0.60 |
| Alb(g/L) (M±SD) | | 36.35±5.47 |
| D-dimer(mg/L) (M,IQR) | | 3.57(2.10) |
| Introperative data | | |
| Operation time(min) (M±SD) | | 97.64±1.90 |
| Blood loss(ml) (M±SD) | | 129.32±5.90 |
| Blood transfusion(n,%) | | 192(22.02%) |
| ICU admission(n,%) | | 360(41.28%) |
| Outcomes(n,%) | | |
| MACCEs | | 112(12.84%) |
| MI | | 34(3.90%) |
| Cardiac arrest | | 6(0.69%) |
| Cardiac shock | | 26(2.98%) |
| Stroke | | 26(2.98%) |
| Death | | 46(5.28%) |
| Total inpatient costs(USD) (M±SD) | | 6372±156 |
| In hospital(days) (M±SD) | |  |
| Totle | | 18.36±14.53 |
| After surgery | | 13.29±11.97 |

COPD, Chronic Obstructive Pulmonary Disease; PE, Pulmonary embolism; NLR, Neutrophil-to-lymphocyte Ratio; MACCEs, Major Adverse Cardiovascular and Cerebrovascular Events; MI, Myocardial Infarction

**Supplementary Table 2.** ROC curve parameters of measurement data.

| Indictors | AUC | Cutoff | P |
| --- | --- | --- | --- |
| Age(years) | 0.492 | 90.5 | 0.797 |
| BI score | 0.460 | 17.5 | 0.167 |
| RCRIscore | 0.511 | 2.5 | 0.711 |
| Preoperative lab results | | | |
| WBC(10^3^/uL) | 0.540 | 7.8 | 0.174 |
| NLR | 0.651 | 11.2 | ＜0.001 |
| Hb(g/L) | 0.620 | 111.5 | ＜0.001 |
| Plt(10^3^/uL) | 0.507 | 258 | 0.821 |
| Na(mmol/L) | 0.567 | 140.4 | 0.021 |
| K(mmol/L) | 0.504 | 3.6 | 0.885 |
| Alb(g/L) | 0.650 | 34.7 | ＜0.001 |
| D-dimer(mg/L) | 0.598 | 3.6 | 0.001 |
| Introperative data | | | |
| Operation time(min) | 0.547 | 104 | 0.108 |
| Blood loss(ml) | 0.566 | 110 | 0.023 |
| In hospital(days) | | | |
| Totle | 0.516 | 20.5 | 0.574 |
| After surgery | 0.534 | 11.5 | 0.242 |

NLR, Neutrophil-to-lymphocyte Ratio

**Supplementary Table 3.** Univariate Analysis of Postoperative MACCEs in Training Group.

| Indictors | MACCEs(n=75) | NMACCEs(n=348) | P |
| --- | --- | --- | --- |
| Male(n,%) | 32(42.7%) | 153(43.9%) | 0.837 |
| ASA grade |  |  | 0.086 |
| II | 2(2.7%) | 20(5.7%) |  |
| III | 45(60.0%) | 237(68.1%) |  |
| IV | 25(33.3%) | 88(25.2%) |  |
| V | 3(4.0%) | 3(0.8%) |  |
| Emergency | 28(37.3%) | 74(21.2%) | 0.003 |
| Hypertension | 38(50.6%) | 177(50.8%) | 0.976 |
| Coronary heart disease | 36(48.0%) | 150(43.1%) | 0.438 |
| Heart failure | 15(20.0%) | 39(11.2%) | 0.038 |
| Cardiac arhythmia | 44(58.6%) | 145(41.6%) | 0.007 |
| Diebete | 9(12.0%) | 52(14.9%) | 0.511 |
| COPD | 12(16.0%) | 55(15.8%) | 0.966 |
| Asthma | 1(1.3%) | 4(1.1%) | 0.894 |
| Pneumonia | 19(25.3%) | 90(25.8%) | 0.924 |
| Bronchiectasis | 14(18.6%) | 67(19.2%) | 0.907 |
| Dementia | 11(14.6%) | 27(7.7%) | 0.058 |
| Seizure | 2(2.6%) | 3(0.8%) | 0.190 |
| Stroke | 22(29.3%) | 112(32.1%) | 0.630 |
| Kidney disease | 14(18.6%) | 47(13.5%) | 0.249 |
| NLR≥11.2 | 56(74.7%) | 168(48.3%) | ＜0.001 |
| Hb≤112(g/L) | 54(72.0%) | 197(56.6%) | 0.014 |
| Na≤140(mmol/L) | 54(72.0%) | 212(60.9%) | 0.072 |
| D-dimer≥3.6(mg/L) | 37(49.3%) | 95(27.3%) | ＜0.001 |
| Alb≤34.7(g/L) | 41(54.7%) | 140(40.2%) | 0.022 |
| Blood loss≥110(ml) | 44(58.7%) | 172(49.4%) | 0.146 |
| Blood transfusion | 36(48.0%) | 133(38.2%) | 0.117 |
| ICU admission | 61(81.3%) | 169(48.5%) | ＜0.001 |
| Total inpatient costs(RMB) (M±SD) | 48560.01±39509.69 | 45977.25±32634.61 | 0.511 |
| In hospital(days) (M±SD) |  |  |  |
| Totle | 18.54±12.71 | 18.30±14.77 | 0.854 |
| After surgery | 14.03±11.22 | 13.15±12.06 | 0.447 |

COPD, Chronic Obstructive Pulmonary Disease; NLR, Neutrophil-to-lymphocyte Ratio

**Supplementary Table 4.** 10-fold cross-validation for the risk prediction model of MACCEs.

| Number | area under the ROC curve |
| --- | --- |
| 1 | 0.951 |
| 2 | 0.713 |
| 3 | 0.702 |
| 4 | 0.687 |
| 5 | 0.825 |
| 6 | 0.807 |
| 7  8  9  10  average | 0.752  0.824  0.760  0.816  0.784 |

**2 Supplementary Figures**

**
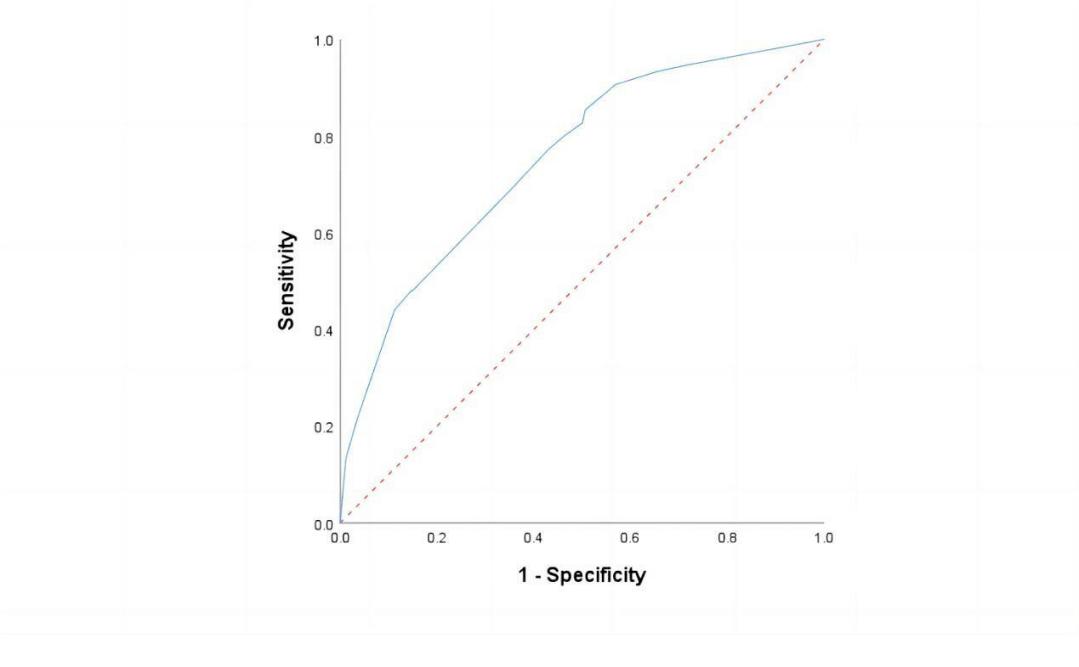
**

**Supplementary Figure 1.** ROC curve for the risk prediction model of postoperative MACCEs in the training group.


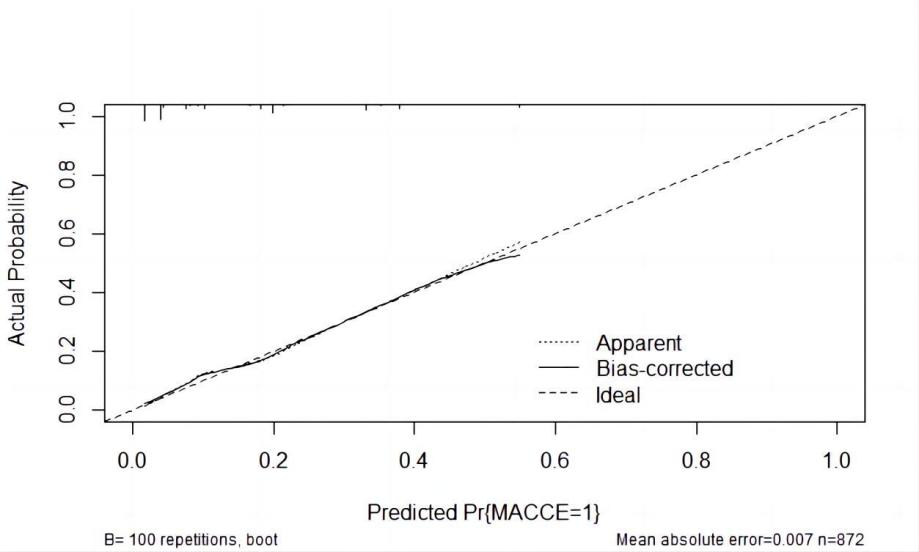


**Supplementary Figure 2.** Calibration curve for MACCEs after non-cardiac surgery in super-elderly patients under general anesthesia.

**
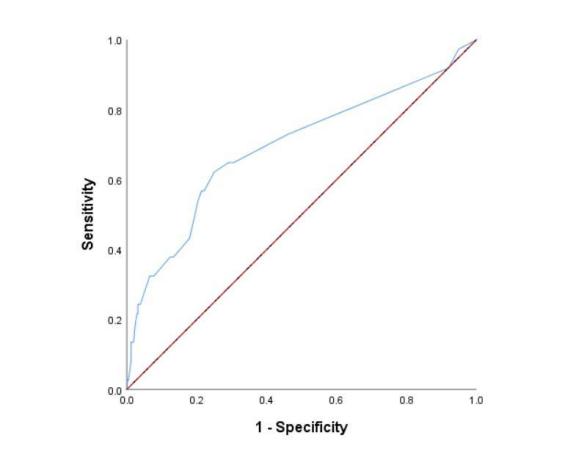
**

**Supplementary Figure 3.** ROC curve for RCRI score in the validation group.

**
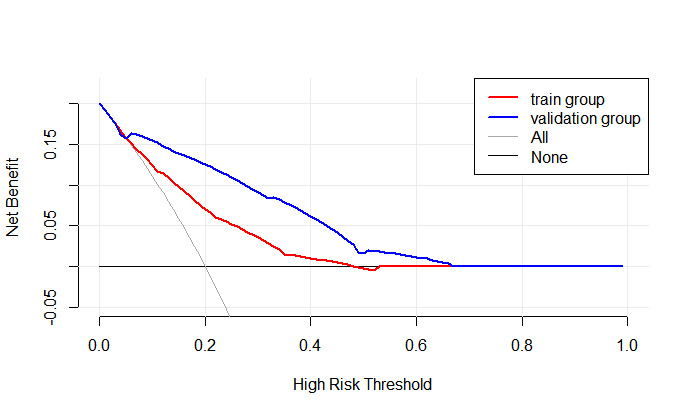
**

**Supplementary Figure 4.** Decision curve analysis for the predictive model of in-hospital postoperative MACCE in patients aged 90 and older.


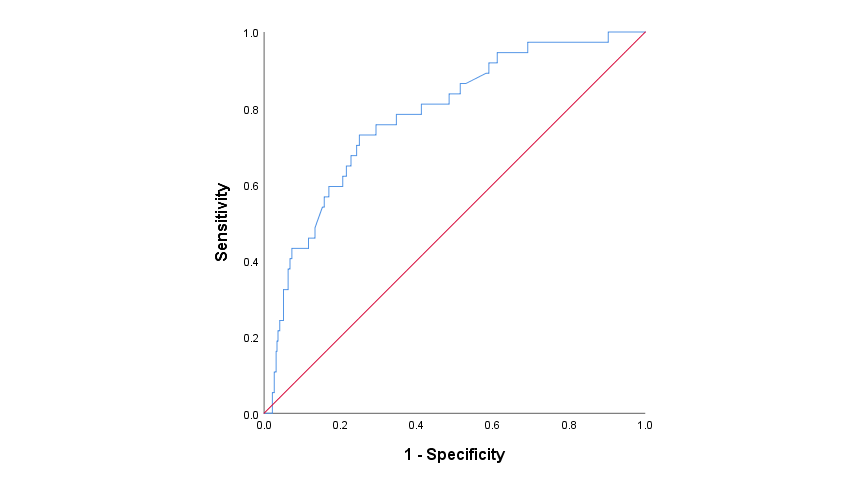


**Supplementary Figure 5.** ROC curve for the risk prediction model B of postoperative MACCEs in the validation group.
